# The double-edged sword role of tumor-associated macrophages: preventing or causing resistance to immunotherapy

**DOI:** 10.1186/s13046-026-03676-9

**Published:** 2026-03-18

**Authors:** Raheleh Roudi, Behnaz Beikzadeh, Babak Beikzadeh, Sima Kalantari, Navid Sobhani

**Affiliations:** 1 Department of Radiology, Molecular Imaging Program at Stanford, Stanford University, 725 Welch Road, Stanford, CA 94305 USA; 2 Department of Molecular Genetics, Faculty of Biological Sciences, Tarbiat Modarres University, Tehran, Iran; 3 Department of Cell and Molecular Biology & Microbiology, Faculty of Biological Science and Technology, University of Isfahan, Isfahan, Iran; 4 Department of Molecular Imaging, Faculty of Advanced Technologies in Medicine, Iran University of Medical Sciences (IUMS), Tehran, Iran; 5 Department of Leukemia, The University of Texas MD Anderson Cancer Center, Houston, TX 77030 USA

**Keywords:** Tumor-associated macrophages, Immunotherapy, Tumor microenvironment

## Abstract

Immunotherapy has transformed cancer treatment, yet only a subset of patients achieves durable responses. Although macrophages play a key role in modulating immune response and could be used to enhance immunotherapy response, accumulating evidence highlights tumor-associated macrophages (TAMs) as both preventors or mediators of cancer, and its related therapies. TAMs contribute to resistance through multiple mechanisms, such as promoting T-cell exclusion, suppressing antitumor immunity via immunoregulatory cytokines, engaging metabolic checkpoints, and facilitating tumor angiogenesis. Clinical and single-cell transcriptomic studies further underscore the association between high TAM infiltration and poor immunotherapy outcomes. Emerging therapeutic strategies aim to reprogram, deplete, or block recruitment of TAMs, with several approaches currently being evaluated in early-phase clinical trials. This review summarizes the double-edged sword of TAMs M1-like proinflammatory or M2-like immunosuppressive states, in causing or preventing cancer. A deeper understanding of TAM heterogeneity and dynamics may enable the rational design of combination therapies and the development of predictive biomarkers to guide personalized immunotherapy.

## Introduction

Over the past decade, immunotherapy has transformed the therapeutic landscape of cancer. Unlike conventional cytotoxic or targeted therapies that act directly on tumor cells, immunotherapy harnesses the host immune system to recognize and destroy malignant cells. Among the most transformative advances are immune checkpoint inhibitors (ICIs) targeting programmed cell death protein-1 (PD-1), its ligand PD-L1, and cytotoxic T-lymphocyte–associated protein-4 (CTLA-4) [1, 2]. By blocking inhibitory pathways that restrain T-cell activity, these agents have yielded durable responses and prolonged survival in several malignancies, including melanoma, non–small cell lung cancer, and renal cell carcinoma [3, 4]. Concurrently, adoptive cell therapies—most notably chimeric antigen receptor–T-cell (CAR-T) therapy have achieved remarkable efficacy in hematologic malignancies, inducing long-term remission in patients with relapsed or refractory B-cell leukemias and lymphomas [5]. Together, these breakthroughs have established immunotherapy as a major pillar of modern cancer treatment.

Despite the successes of both ICIs and adoptive cell therapies, a large proportion of patients with solid tumors either do not respond initially (primary resistance) or have a relapse after an initial response (acquired resistance) [6, 7]. Resistance to ICIs is driven by a complex interplay of tumor-intrinsic mechanisms, such as impaired antigen presentation, oncogenic signaling, and interferon pathway defects, and tumor-extrinsic mechanisms residing within the tumor microenvironment (TME) [8]. The TME is a heterogeneous and dynamic ecosystem composed of malignant cells, stromal components, extracellular matrix (ECM), and a spectrum of infiltrating immune cells that collectively shape tumor progression and therapy response [9].

TAMs are a component of the TME. They play a central and multifaceted role in regulating tumor immunity. Macrophages display remarkable plasticity, polarizing toward either M1-like proinflammatory or M2-like immunosuppressive states in response to environmental cues, like a double-edged sword [10]. In most solid tumors, TAMs are skewed toward an M2-like phenotype under the influence of cytokines and metabolic factors such as interleukin (IL)-10, transforming growth factor (TGF)-β, colony-stimulating factor (CSF)-1, and hypoxia [11, 12]. These TAMs secrete immunoregulatory molecules (e.g., IL-10, arginase-1, prostaglandin E2), express checkpoint ligands (PD-L1, B7-H4), and suppress cytotoxic T-cell activation [13–16]. Beyond immune suppression, TAMs also promote angiogenesis, matrix remodeling, epithelial–mesenchymal transition, and metastasis [12]. Elevated TAM infiltration correlates with poor prognosis and reduced responsiveness to ICIs in melanoma, lung cancer, and pancreatic cancer [17]. In this review, we will delve into TAM biology, discussing their double-edged sword characteristics, and explain novel therapies targeting TAMs, with the aim to restore their antitumor functions.

## Biology and heterogeneity of TAMs

### Origins

Cancer progression occurs within the TME [18, 19]. TAMs represent the most abundant immune cell population within the TME of most solid tumors, often comprising a significant proportion of the total tumor mass [19, 20]. These versatile immune cells exhibit remarkable phenotypic plasticity, capable of adopting either tumor-suppressive or tumor-promoting functions depending on local microenvironmental signals and spatial distribution [21, 22].

The diverse nature of TAMs stems from their distinct origins [23]. One major group, the tissue-resident macrophages, originates during embryonic development from precursors in the yolk sac and fetal liver. These cells populate tissues early in life and can maintain their population through in situ self-renewal, independent of circulating precursors [24, 25]. In contrast, the other group, monocyte-derived macrophages, arises from hematopoietic stem cells in the adult bone marrow [26]. These cells circulate in the blood as monocytes before being recruited into tissues in response to various signals, including inflammation associated with cancer [27]. Evidence suggests that tissue-resident macrophages are typically involved in the initial phases of tumor formation [28]. As the tumor grows, it increasingly recruits monocyte-derived macrophages, which are more prone to adopting the pro-tumor phenotype that facilitates disease progression and metastasis [29].

TAMs in pancreatic ductal adenocarcinoma (PDAC) arise from two distinct ontogenies: circulating monocytes and embryonically derived tissue-resident macrophages that persist in the adult pancreas and expand locally by self-renewal [30]. These populations are functionally specialized, with monocyte-derived TAMs enriched for antigen presentation and inflammatory pathways, whereas tissue-resident macrophages exhibit a dominant pro-fibrotic, stromal-remodeling program that promotes desmoplasia and tumor progression [30]. Resident macrophage–driven fibrosis is highly context dependent: it supports tissue repair in pancreatic injury but becomes tumor promoting in PDAC by creating a stiff, immune-excluded microenvironment [31]. Within this resident compartment, a proliferative subset displays metabolic reprogramming that limits gemcitabine efficacy through altered pyrimidine metabolism, thereby driving chemoresistance [32]. Together, these findings establish macrophage ontogeny as a key determinant of TAM function and highlight resident macrophages as critical therapeutic targets in PDAC.

Several therapeutic strategies have been developed to preferentially impact monocyte-derived TAMs by either inhibiting their recruitment, depleting them, or altering their functional polarization in the TEM. Inhibition of the CSF-1/CSF-1R axis with small-molecule inhibitors (e.g., BLZ945, PLX3397/pexidartinib) or monoclonal antibodies (e.g., emactuzumab) reduces TAM numbers and limits the differentiation and survival of recruited monocyte-derived macrophages, often improving effector T-cell infiltration and anti-tumor immune responses in preclinical models and clinical settings when combined with other immunotherapies [33–36]. Targeting monocyte recruitment through the CCL2/CCR2 signaling axis using antagonists (e.g., PF-04136309) has shown efficacy in preclinical models by blocking trafficking of CCR2 + monocytes from circulation into tumors, thereby reducing TAM accumulation [37]. Cytotoxic chemotherapeutics such as trabectedin induce apoptosis in circulating monocytes and macrophages, leading to reduced TAM density and lower CCL2 levels in tumor tissue, representing another mechanism to preferentially deplete monocyte-derived TAMs [38]. Additional approaches include bisphosphonates and nanomedicine strategies that either deplete macrophages broadly or inhibit monocyte infiltration into tumors [39, 40]. While many of these agents do not exclusively differentiate between monocyte-derived versus tissue-resident macrophages, their mechanistic emphasis on blocking monocyte recruitment and survival implies preferential modulation of recruited TAM populations, underscoring the importance of further defining and exploiting TAM ontogeny in therapeutic design.

### Dual functionality of TAMs

The traditional classification of TAMs into a rigid antitumor (M1) and pro-tumor (M2) framework is now considered an oversimplification of their true biological complexity [41]. Although anti-tumor and pro-tumorigenic profiles are still described in TAMs, these cells, rather than existing as two distinct and stable populations, display a remarkable ability to shift their characteristics along a continuous spectrum from anti-tumor to pro-tumorigenic, adapting their functions in response to dynamic signals within the TME [42]. This functional and phenotypic adaptability means that TAMs are not confined to a binary system but represent a range of activation states with diverse and sometimes overlapping roles [43]. A summary of various TAM subsets is shown in Table 1.

**Table 1:** Human Tumor-Associated Macrophage (TAM) subsets defined by single-cell transcriptomics and functional states

TAM Subset (Human-relevant)	Key Transcriptional / Protein Markers	Dominant Functional Programs	Clinical / Therapeutic Relevance
TREM2⁺ lipid-associated TAMs (LAMs)	TREM2, APOE, LILRB1, LGALS3, FABP5, CTSB	Lipid metabolism, immunosuppression, T-cell exclusion	Associated with resistance to ICIs; enriched in non-responders; targetable via TREM2 or metabolic reprogramming
SPP1⁺ (osteopontin⁺) TAMs	SPP1, MERTK, MARCO, CTSK, VCAN	ECM remodeling, angiogenesis, stromal crosstalk	Linked to fibrosis, metastasis, immune exclusion, and therapy resistance
Interferon-responsive TAMs (IFN-TAMs)	IFIT1, IFIT3, ISG15, STAT1, CXCL9, CXCL10	Antigen presentation, inflammatory signaling, T-cell recruitment	Associated with favorable responses to ICIs
CXCR4⁺ hypoxia-associated TAMs	CXCR4, VEGFA, HIF1A, CA9, SLC2A1	Hypoxia adaptation, angiogenesis, immune suppression	Promote immune exclusion and resistance; enriched post-therapy
CCR2⁺ monocyte-derived TAMs	CCR2, LYZ, FCN1, S100A8/A9 (early)	Rapid recruitment, suppressive differentiation	Drive myeloid rebound and therapy-induced resistance
Resident tissue-derived TAMs	FOLR2, LYVE1, TIMD4, CD163 (context-dependent)	Tissue remodeling, niche maintenance, fibrosis	Often resistant to CSF-1R depletion; contribute to stromal barriers
Antigen-presenting TAMs (AP-TAMs)	HLA-DRA, HLA-DRB1, CD74, CIITA	Antigen presentation, T-cell priming	May support immunotherapy responses in selected tumors
Metabolically reprogrammed TAMs	ARG1, IDO1, CPT1A, SLC16A3 (MCT4)	Arginine depletion, lipid oxidation, lactate sensing	Stabilize T-cell exhaustion; limit ICI reinvigoration
FcγR-high TAMs	FCGR1A, FCGR2A, FCGR3A	Antibody uptake, immune complex handling	Sequester ICIs and reduce antibody bioavailability
Proliferative TAMs	MKI67, TOP2A, STMN1	Local self-renewal, niche persistence	Maintain suppressive TAM pools independent of monocyte input

TAM subsets are defined by transcriptional and functional programs rather than fixed polarization states. These populations exhibit high plasticity and may coexist or transition within the same tumor microenvironment

#### Anticancer activities of TAMs

Under defined situations, TAMs can effectively halt malignancy and boost treatment responses [18]. When these immune cells become polarized towards the anti-tumor profile— typically as a result of stimuli such as interferon-γ, bacterial products like lipopolysaccharide, or ligands for Toll-like receptors—they display a protective immunological role in neoplastic diseases [44]. M1-like TAMs contribute to cancer suppression by executing a broad range of actions, including engulfing tumorous cells through phagocytosis, initiating antibody-driven cellular cytotoxicity by acting as antigen-presenting cells, and orchestrating direct killing mechanisms of the adaptive immune system [25, 45, 46]. Additionally, the production of molecules like reactive oxygen species and nitric oxide in M1-like TAMs is a crucial trigger for programmed death of cancer cells [47]. M1-like TAMs can also secrete pro-inflammatory cytokines, such as IL-12, tumor necrosis factor (TNF)-α, and IFN-γ, which play pivotal parts in activating NK cells and cytotoxic T-lymphocytes, thereby strengthening antitumor defense systems [48, 49]. M1-like TAMs act as antigen-presenting cells by elevating the expression of MHC class II and co-stimulatory molecules, which enables robust activation and maintenance of T-cell responses against malignancies [50].

Evidence from specific cancer types underscores the context-dependent beneficial impact of TAMs [51]. For instance, colorectal cancer specimens often show a paradoxical link between high TAM presence and improved clinical outcomes, marked by greater numbers of CD8 + T cells, reduced spread of disease, and longer survival rates [52–54]. A unique subtype, TREM2 + multinucleated macrophages, has been identified in head and neck squamous cell carcinoma, where these cells, demonstrating signs of a foreign body reaction, are associated with superior prognoses, notably in those receiving chemotherapy before surgery [55]. By clearing extracellular clusters of keratin and expressing enzymes like chitinase, these giant macrophages appear to be involved in the preservation of tissue integrity and immune monitoring rather than in cancer promotion [55]. Newly published data spotlight ZEB1-expressing macrophages as key players in mobilizing and activating cytotoxic T cells, offering intriguing avenues for the manipulation of macrophage biology for clinical benefit [56, 57].

At the boundary between the tumor mass and the surrounding stromal tissue, TAMs can establish suppressive barriers [46], both physical and functional, to immune attack [58]. These TAMs are capable of remodeling the local environment to create chemokine gradients that actively prevent cytotoxic T cells from infiltrating the tumor core [59]. This formation of an immune-exclusionary barrier by TAMs at the tumor’s edge is a significant mechanism of resistance to immunotherapies that rely on T-cell–mediated killing [60].

#### Pro-tumor and immunosuppressive features of TAMs

The TME can corrupt M1-like macrophages, causing them to polarize toward a more pro-tumor state (M2-like) [61]. These M2-like TAMs (like SPP1 + TAMs) actively support the tumor by promoting angiogenesis [62], migration, ECM remodeling [63], EMT [64, 65], and immunosuppression that drives therapeutic resistance [62, 63, 66–70]. Across most solid tumor types, the predominant macrophage phenotype reflects pro-tumorigenic polarization [18].

M1-like macrophages are typically polarized through the lipopolysaccharide/IFN-γ axis, whereas pro-tumorigenic macrophages are induced via the IL-4/13/10 polarization pathway [71, 72]. Furthermore, noncoding RNAs play a role in regulating TAM polarization. Tumor-associated heterogeneity is evident in functional disparities among macrophages from diverse origins, spatial localization differences within a single tumor, or variations across distinct tumor types [68].

Clinical investigations have shown that increased TAM infiltration is associated with unfavorable prognosis in numerous human tumors [73], especially breast, colorectal, and cervical cancers [74–76]. In contrast, certain studies indicate that TAM infiltration is closely associated with improved prognosis in ovarian and colorectal cancers [68, 77], underscoring the variable relationship between TAM infiltration and clinicopathologic features in distinct TAM subtypes [78].

Factors secreted by the TME, such as IL-4, IL-10, TGF-β, and hypoxia-inducible factor (HIF)-1α, orchestrate the activation of transcriptional networks, including STAT3, STAT6, and IRF4, all essential for the development of this pro-tumoral state [79, 80].

Impaired antitumor immunity is a consequence of TAM activity through several mechanisms [81]. M2-like TAMs express checkpoint proteins such as PD-L1, PD-L2, and CD47—that diminish T-cell activity and phagocytosis through direct inhibitory interactions [82–84]. They recruit Tregs through chemokine gradients [85]. Moreover, TREM2 + TAMs create a steric hindrance, physically obstructing T-cell entry in tumors, reducing the efficacy of immunotherapy [46, 69, 86–88].

Clinical studies confirm a consistent association between increased infiltration of TAMs exhibiting M2-like or CD163 + phenotypes and adverse prognoses in a wide spectrum of cancers, with negative impacts noted in lung, breast, gastric, bladder, and pancreatic malignancies [89–91]. In breast carcinoma, TAM density in the surrounding stroma has emerged as a more potent indicator of negative outcome than intratumoral TAM numbers; equivalently, similar roles of stromal TAM density have been documented in glioblastoma, hepatocellular carcinoma, and non–small cell lung cancers [91, 92].

With the aid of single-cell RNA sequencing discoveries, distinctive TAM populations including TREM2+, SPP1+, MARCO+, SIGLEC10+, and CCL18 + subtypes—have been discovered to be implicated in furthering both immunosuppression and resistance to monotherapies and combination therapies [91, 93–95].

Finally, single-cell RNA sequencing analyses have identified other TAM populations with strong IFN response gene signatures, which do not fit neatly into the anti-tumor or pro-tumorigenic paradigm [62, 96, 97]. Although not fully cytotoxic like classic anti-tumor cells, these interferon-responsive TAMs can exhibit some antitumor capabilities and are thought to be crucial for orchestrating successful responses to ICIs [98]. Type I and type II interferons are critical signaling molecules, often by inducing significant metabolic alterations within the macrophages and fostering a synergistic relationship with T cells to enhance anticancer immunity [99]. Of note, the presence of IFN-primed macrophages in the TME is increasingly being recognized as a favorable biomarker for immunotherapy [70, 100].

In addition, single-cell RNA sequencing studies demonstrated that TREM2⁺ TAMs have emerged as a recurrent and conserved subset across multiple cancer types, defined by enrichment of genes associated with immunosuppression, lipid metabolism, and extracellular matrix remodeling. These cells correlate with reduced effector T-cell activity and poor clinical outcomes, and are considered pro-tumorigenic in both human samples and preclinical models [101, 102]. In parallel, SPP1⁺ macrophages represent another distinct TAM population identified across tumor types, characterized by high expression of secreted phosphoprotein 1 (osteopontin) and enriched in hypoxic and stromal niches [103]. These SPP1⁺ TAMs contribute to angiogenesis, extracellular matrix remodeling, and immunosuppression by interacting with cancer-associated fibroblasts and impairing CD8⁺ T-cell recruitment, and have been linked to adverse clinical features and therapy resistance [104]. Both TREM2⁺ and SPP1⁺ subsets frequently co-express lipid-metabolic and scavenger receptors (e.g., FABP5, APOE, CD36), reflecting their lipid-associated macrophage (LAM) phenotype and highlighting distinct metabolic programs that drive functional specialization in the tumor microenvironment, distinct from canonical M-polarization states [105]. Integrating these transcriptionally defined subsets into our mechanistic framework thus provides a more accurate and contemporary picture of TAM functional heterogeneity, replacing broad “M2-like” categories with subset-specific roles that are supported by high-dimensional profiling across cancers.

##### Promotion of tumor growth and angiogenesis

One of the most well-established pro-tumorigenic roles of TAMs is their ability to orchestrate angiogenesis, thereby supporting tumor growth and metastasis. TAMs preferentially localize to hypoxic tumor regions, where stabilization of HIF-1α and HIF-2α upregulates the transcription of angiogenic mediators, most notably vascular endothelial growth factor (VEGF) [8, 106, 107]. VEGF stimulates endothelial cell proliferation, migration, and survival, while also increasing vascular permeability, ultimately leading to the formation of the aberrant, tortuous, and leaky vasculature characteristic of advanced tumors [8, 106, 107].

A specialized subset of TIE2-expressing TAMs is particularly enriched in perivascular and hypoxic regions, where these TAMs secrete VEGF-A and platelet-derived growth factor β (PDGF-β), reinforcing localized angiogenesis [108]. PDGF recruits pericytes and smooth vascular muscle cells to developing vessels. Although this process stabilizes new vasculature under physiological conditions, in tumors it often results in abnormal coverage and disorganized vessel networks, further exacerbating hypoxia and poor perfusion [109, 110].

TAMs also release fibroblast growth factor-2 (FGF-2), which acts synergistically with VEGF to promote endothelial survival, ECM remodeling, and vessel branching [111, 112]. Importantly, TAMs secrete proteolytic enzymes such as matrix metalloproteinases (MMPs) and urokinase-type plasminogen activator, which degrade the ECM, liberating sequestered angiogenic factors (including FGF-2 and VEGF) and thereby amplifying angiogenic signaling [113].

The combined actions of VEGF, PDGF, and FGF establish a TME that sustains oxygen and nutrient delivery, while simultaneously facilitating tumor cell intravasation and metastatic dissemination. Aberrant TAM-mediated angiogenesis also contributes to therapeutic resistance, because abnormal vascular networks impair the delivery of chemotherapeutics and immune effector cells, while persistent hypoxia drives immune suppression and selection of more aggressive tumor phenotypes [8, 106, 110].

Therapeutically, strategies to block TAM-derived angiogenic signals are being actively explored. These strategies include VEGF neutralization (e.g., bevacizumab), inhibition of CSF-1 receptor (CSF-1R) to deplete or reprogram TAMs, and combined anti-angiogenic and immunotherapeutic approaches aimed at vascular normalization and restoration of treatment responsiveness [110, 111].

##### Hypoxia and lactate accumulation

**Fig. 1:**
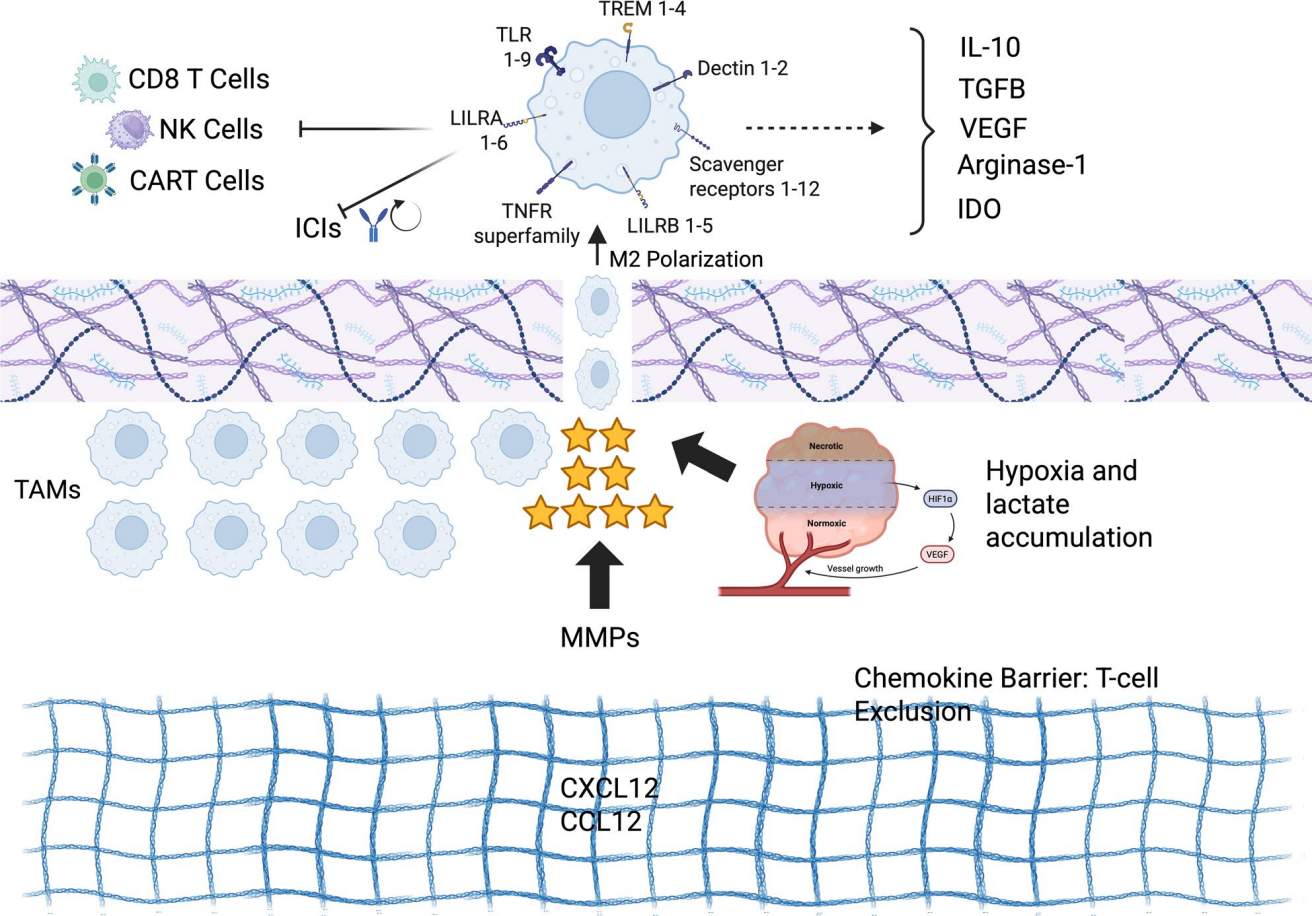
Immune suppressive and tumor microenvironment-driven polarization functions of Tumor Associated Macrophages (TAMs). Tumor microenvironment cues, enshrining hypoxia, lactate accumulation and matrix remodeling mediated by matrix metalloproteinases (MMPs), promote polarization of TAMs toward an M2-like immunosuppressive state within the extracellular matrix. The subsequent polarized TAMs inhibit antitumor immune responses by limiting activity of CD8+ T cells activity, natural killer (NK) cells, and CAR T cells, and reducing responsiveness of Immune Checkpoint Inhibitors (ICIs). These interactions are mediated by the production of immunosuppressive and pro-tumorigenic factors, entailing arginase-1, indoleamine 2,3-dioxygenase (IDO), IL-10, VEGF and TGF-β

Regions of hypoxia, common in poorly vascularized tumors, stabilize hypoxia-inducible factors (HIF1α and HIF2α), initiating transcriptional programs that favor pro-tumorigenic polarization and angiogenic factor release [114]. Therefore, HIF-1α and HIF-2α are master regulators of the macrophage phenotype [50]. In parallel, cancer cell–derived lactate, a major glycolytic byproduct, acts not only as a metabolite but also as a signaling molecule. Through the MCT–HIF-1α axis and activation of ERK/STAT3 and mTOR pathways, lactate induces macrophages to upregulate CD163, arginase-1 (ARG1), and other M2-associated markers [115]. Beyond signaling, lactate directly rewires macrophage epigenetics by driving histone lysine lactylation (Kla), reinforcing expression of immunoregulatory genes [116]. Together, hypoxia and lactate establish a metabolic niche that locks TAMs into a suppressive state (Fig. 1).

Superimposed on hypoxia- and lactate-driven cues, TAMs undergo extensive lipid uptake through scavenger receptors including CD36 and TREM2, leading to intracellular accumulation of fatty acids, oxidized lipoproteins, and apoptotic tumor debris [117]. This lipid overload promotes lipid peroxidation and reactive oxygen species generation, reinforcing protumor macrophage programs while depleting essential metabolic substrates within the tumor microenvironment [117]. Oxidized lipid species and lipid peroxidation byproducts are subsequently released or transferred to infiltrating T cells via paracrine signaling and extracellular vesicles, where they disrupt mitochondrial integrity and induce endoplasmic reticulum stress [118]. Activation of stress-response pathways such as the PERK–eIF2α–ATF4 axis in T cells directly drives transcriptional upregulation of exhaustion-associated receptors, including PD-1, TIM-3, and LAG-3, while constraining glycolytic capacity and mitochondrial fitness required for sustained effector function. In parallel, lipid-induced activation of STAT3 and NF-κB signaling in TAMs sustains high-level expression of inhibitory ligands such as PD-L1 and B7-H4, reinforcing chronic checkpoint engagement independently of antigen load [118]. Together, these hypoxia-, lactate-, and lipid-driven metabolic programs converge to impose persistent metabolic stress and exhaustion signaling in T cells, mechanistically locking the tumor microenvironment into a state of durable immune dysfunction that is intrinsically resistant to immune checkpoint blockade.

##### Nutrient competition with T cells

TAMs actively deprive effector T cells of essential nutrients required for clonal expansion and cytotoxic activity. High expression of ARG1 depletes arginine, while indoleamine 2,3-dioxygenase (IDO) catabolizes tryptophan, producing kynurenine metabolites that suppress T-cell receptor signaling. Moreover, TAMs consume glucose through aerobic glycolysis, leaving effector T cells metabolically starved. This nutrient scarcity activates GCN2 kinase and dampens mTOR signaling in T cells, leading to impaired proliferation, reduced cytokine production, and eventual functional exhaustion [119].

##### Lipid metabolic reprogramming

The TME imposes profound metabolic pressures that reshape macrophage function toward an M2-like, immunosuppressive phenotype. Beyond amino acids and glucose, TAMs also undergo a profound shift toward lipid-driven metabolism, which supports long-term immunosuppressive function. Lipid droplet accumulation, coupled with enhanced fatty acid oxidation via enzymes such as CPT1A, promotes oxidative phosphorylation and stabilizes M2-like features [120]. In hepatocellular carcinoma, FABP5-mediated fatty acid transport activates PPARγ signaling, reinforcing immunoregulatory gene expression and dampening T-cell responses [121]. Importantly, disrupting fatty acid oxidation or enhancing lipolysis (e.g., via MGLL overexpression) reprograms TAMs toward a pro-inflammatory state, highlighting lipid metabolism as a therapeutic vulnerability [120, 122].

Beyond glucose and amino acid metabolism, TAMs undergo profound lipid metabolic reprogramming enforcing immunosuppression directly. Enhanced uptake of fatty acids through scavenger receptors and lipid transporters leads to the accumulation of lipid droplet and a shift toward fatty acid oxidation and oxidative phosphorylation. This metabolic state supports long-lived suppressive TAM phenotypes and is accompanied by increased generation of lipid peroxidation and reactive oxygen species. Lipid-derived oxidative signals impair macrophage antigen presentation and costimulatory capacity while increasing the release of inflammatory lipid mediators that sustain inhibitory signaling in the tumor microenvironment. Consequently, effector T cells exposed to lipid-reprogrammed TAMs exhibit persistent expression of exhaustion markers such as PD-1, TIM-3, and LAG-3, reduced cytokine production, and diminished responsiveness to immune checkpoint blockade. These findings suggest TAM lipid metabolism as a direct mechanistic driver of T-cell exhaustion and ICI failure, rather than a passive correlate of immune suppression.

##### Immune system suppression

TAMs are key orchestrators of immune evasion in the TME, exerting their suppressive effects through multiple complementary mechanisms, including checkpoint ligand expression, chemokine secretion, and ECM remodeling.

###### Checkpoint ligand expression

TAMs are central contributors to immune suppression in the TME through expression of inhibitory checkpoint ligands. Among these, PD-L1 (CD274) and PD-L2 (PDCD1LG2) are the best characterized. Both engage PD-1 on activated T cells, leading to impaired T-cell receptor signaling, reduced cytokine secretion, diminished cytotoxic activity, and ultimately T-cell exhaustion [123–125].

###### Cytokine-driven induction

PD-L1 expression on TAMs is tightly regulated by immune-derived signals. IFN-γ, released by effector T and NK cells, is the most potent inducer, activating the JAK2–STAT1–IRF1 transcriptional axis, which directly upregulates PD-L1 [126]. In addition, other inflammatory cytokines such as TNF-α and IL-6 can sustain PD-L1 expression via NF-κB–dependent pathways [127].

###### Metabolic and stress signals

Hypoxia, a defining feature of solid tumors, strongly enhances PD-L1 transcription in macrophages through HIF-1α binding to hypoxia-responsive elements in the PD-L1 promoter [128, 129]. In parallel, oncogenic pathways such as phosphoinositide 3-kinase (PI3K)/AKT/mTOR and MAPK/ERK signaling also contribute to sustained PD-L1 expression [130, 131].

###### PD-L2 regulation

Compared with PD-L1, PD-L2 is less studied but increasingly recognized as clinically relevant. Its expression on macrophages is driven primarily by IL-4/IL-13–mediated STAT6 signaling, reflecting Th2-type immune skewing in the TME [132]. Beyond PD-1 binding, PD-L2 interacts with repulsive guidance molecule b (RGMb), a receptor involved in tissue tolerance and T-cell differentiation, suggesting a broader immunomodulatory role than previously appreciated [133, 134].

###### TAM remodeling of the ECM

TAMs play a critical role in shaping the TME to limit effector T-cell infiltration and suppress antitumor immunity. One major mechanism is the secretion of chemokines, including CXCL12 and CCL22. CXCL12 forms chemotactic gradients that retain cytotoxic CD8 + T cells in the tumor periphery, preventing their migration into tumor cores, while also recruiting immunosuppressive populations such as Tregs and myeloid-derived suppressor cells (MDSCs) [135]. Similarly, CCL22, often secreted in conjunction with TAM-derived IL-10 and TGF-β, promotes Treg recruitment via CCR4 signaling, enhancing local immunosuppression and reinforcing immune-privileged niches within the tumor [110, 136]. A distinct perivascular TAM subset characterized as MRC1 + TIE2Hi CXCR4Hi accumulates around blood vessels of breast tumors following doxorubicin chemotherapy, and the accumulation of these cells is thought to promote tumor revascularization and disease recurrence via the VEGF-A signaling pathway. Targeting this TAM population by blocking CXCR4 after chemotherapy markedly reduced revascularization and tumor regrowth, supporting the clinical potential of this strategy [137].

Concurrently, TAMs actively remodel the ECM through the secretion of MMPs, particularly MMP-2 and MMP-9. These enzymes degrade structural ECM proteins such as collagen and fibronectin, dynamically altering stromal architecture and creating physical barriers that impede T-cell infiltration [113, 138]. ECM remodeling also generates biochemical cues, including the release of growth factors and chemokine-binding sites, further modulating immune cell trafficking and function [139]. This dual activity—chemokine-mediated immune cell positioning combined with ECM restructuring—establishes both chemical and physical barriers that effectively sequester effector T cells in the tumor periphery and exclude them from tumor nests. These TAM-driven barriers contribute to the phenomenon of immune “cold” tumors, which are resistant to T-cell–based immunotherapies, including checkpoint inhibitors. Importantly, targeting TAMs or their chemokine/MMP-mediated pathways can enhance T-cell infiltration, restore antitumor immunity, and improve responses to immunotherapy [9, 11, 12]. Accordingly, shifting TAMs toward an immune-supportive phenotype offers a strategy to remodel the immunosuppressive TME and enhance the efficacy of ICI-based therapies; this can be achieved with agents that promote macrophage re-polarization and with nanoparticles engineered to selectively reprogram TAMs toward a reparative, antitumor state [140].

###### Phagocytosis activity of TAMs

In addition to TAM’s roles in stromal remodeling and immune regulation, macrophages possess a potent intrinsic capacity to directly eliminate malignant cells through phagocytosis, which represents a fundamental but frequently suppressed antitumor mechanism [141]. In many cancers, tumor cells evade macrophage engulfment by expressing inhibitory “don’t eat me” signals, most notably through the CD47–signal regulatory protein alpha (SIRPα) axis, which transmits inhibitory signals that block phagocytosis. Blockade of this pathway restores macrophage-mediated tumor cell clearance and enhances antitumor immunity in multiple preclinical models and early clinical studies [142, 143]. Additional phagocytosis checkpoints further restrain macrophage antitumor activity. Tumor cell expression of CD24, which signals through Siglec-10, and MHC class I, which engages LILRB1, also suppresses macrophage engulfment, revealing redundant inhibitory circuits that protect tumor cells from innate immune attack [144]. In contrast, pro-engulfment signals such as calreticulin exposure cooperate with Fc receptor–mediated antibody opsonization to trigger cytoskeletal remodeling and phagosome formation [145].

Within the TME, hypoxia, metabolic stress, and tumor-derived cytokines further suppress macrophage phagocytic capacity and skew macrophages toward an immunosuppressive phenotype [146]. This inhibition not only limits direct tumor cell clearance but also reduces antigen cross-presentation and subsequent T cell activation [147]. Therapeutic strategies aimed at restoring macrophage phagocytosis—through checkpoint blockade, antibody-based opsonization, or macrophage reprogramming—synergize with chemotherapy and immune checkpoint inhibitors to enhance antitumor efficacy in preclinical models.

Together, these findings establish macrophage-mediated phagocytosis as a central effector mechanism of innate antitumor immunity and highlight the therapeutic potential of targeting macrophage immune checkpoints to overcome tumor immune evasion.

##### Interference with therapeutic agents

Beyond shaping the immune TME through checkpoint ligand expression, chemokine secretion, and ECM remodeling, TAMs directly compromise the efficacy of therapeutic agents via multiple mechanisms.

###### Fcγ receptor–mediated antibody clearance

TAMs express high levels of activating Fcγ receptors (FcγRs), including FcγRI, FcγRIIa, and FcγRIII. These receptors recognize the Fc portion of therapeutic monoclonal antibodies, such as anti–PD-1, anti–PD-L1, and anti–CTLA-4, leading to antibody internalization and lysosomal degradation. This process reduces antibody availability for binding to target receptors on T cells or tumor cells, thereby blunting therapeutic efficacy [124, 148, 149]. In vivo imaging studies have shown that TAMs can rapidly sequester anti–PD-1 antibodies, effectively creating a “sink” that limits T-cell reinvigoration [148]. FcγR-mediated uptake is particularly relevant in tumors with high macrophage density, and it can also modulate antibody-dependent cellular cytotoxicity, potentially reducing therapeutic potency.

###### Suppression of CAR-T and other adoptive cell therapies

TAMs impede the efficacy of CAR-T and other adoptive cell therapies through a combination of both paracrine immunosuppression and physical exclusion mechanisms. TAMs secrete cytokines IL-10, TGF-β, and prostaglandin E2 that dampen T-cell proliferation and cytotoxic function [150, 151]. Additionally, TAMs produce reactive oxygen species and reactive nitrogen species that induce T-cell exhaustion and apoptosis [150, 152]. Beyond biochemical suppression, TAMs remodel the ECM via MMPs, creating physical obstacles that obstruct CAR T-cell infiltration into tumor cores [138].

TAMs expressing PD-L1 and PD-L2 can engage PD-1 on CAR T cells, directly suppressing their activity within the TME [151, 153]. Collectively, these mechanisms position TAMs as a major hurdle to both antibody- and cell-based immunotherapies, particularly in “immune-excluded” or “cold” tumors characterized by dense myeloid infiltration. Therapeutic strategies that deplete or reprogram TAMs, block FcγR-mediated antibody sequestration, or modulate TAM-derived cytokines and ECM remodeling are emerging as promising combinatorial approaches to enhance immunotherapy efficacy [9, 11, 12].

Macrophages sensitivity to therapy varies [68]. In some tumors, chemoresistance has been reproducibly linked to the accumulation of insensitive TAM subsets, including CD45 + CD11b+ CD14 + macrophages, CD11b+ Ly6C+ TIE2 + macrophages, and MRC1 + TIE2HiCXCR4Hi macrophages. Notably, post-chemotherapy targeting of these accumulated TAM populations substantially mitigates tumor regrowth and metastatic dissemination [68, 137, 154].

Consequently, reprograming TAMs to shift from pro-tumorigenic to tumoricidal phenotypes may promote tumor regression and achieve favorable patient outcomes Collectively, these findings implicate distinct macrophage subpopulations in modulating treatment efficacy and highlight the potential of selectively targeting insensitive TAMs as an innovative therapeutic strategy to prevent resistance. Thus, there exists an urgent need to investigate TAM heterogeneity and develop targeted interventions against specific TAM subsets [68].

Dynamic alterations in TAM subpopulations occur during tumor development and correlate with immunotherapy efficacy [123–126], underscoring the need to better define TAM heterogeneity and its contribution to therapeutic response [127, 128]. Accumulating evidence identifies TAMs as key mediators of immunotherapy resistance, although the underlying mechanisms remain incompletely understood. Current TAM-targeting strategies include blockade of pro-tumor functions, inhibition of immune checkpoint signaling, prevention of monocyte recruitment, enhancement of macrophage phagocytosis, and reprogramming of TAMs toward an immunostimulatory phenotype [46].

Given their central role in shaping responses to ICI therapy, TAMs must be evaluated within the context of ICI-based regimens. A reciprocal interaction between TAMs and regulatory T cells (Tregs) promotes immune evasion and ICI resistance [129]. TAMs recruit Tregs into the TME through chemokine- and cytokine-mediated mechanisms [130–132], while TAM-derived factors contribute to the generation of inducible Tregs from CD4⁺ CD25⁻ T cells. In turn, Tregs further reinforce the immunosuppressive phenotype of TAMs [133]. TAMs also directly limit ICI efficacy [116, 134] by expressing checkpoint ligands, including PD-L1, PD-L2, CD80, CD86, and VISTA, which can sequester ICIs such as anti–PD-L1 antibodies or competitively bind anti–PD-1 antibodies, reducing their availability to effector T cells [116]. Accordingly, combination strategies targeting TAM function alongside ICIs are under active investigation; notably, anti-CD40 combined with anti-CSF-1R antibodies has been shown to convert immunologically “cold” tumors into “hot” tumors, enhancing T-cell infiltration and antitumor activity [135].

Adoptive cell transfer therapies, including CAR-T, have shown substantial efficacy in hematologic malignancies but remain less effective in solid tumors due to tumor heterogeneity and barriers imposed by the TME [136]. Effective cytotoxic activity requires infiltration of transferred cells into tumor sites, a process often hindered by abnormal tumor vasculature and limited vascular access [137]. A TAM subpopulation expressing TIE2 in circulation or within the TME is closely associated with intratumoral neovascularization [138]. In this context, Chimeric antigen receptor macrophages (CAR-M) have emerged as a promising alternative, as they can more efficiently infiltrate solid tumors and actively remodel the immunosuppressive microenvironment to an M1 phenotype by producing proinflammatory cytokines [139]. A variety of tumor-associated antigens have been targeted by CAR-M, including carcinoembryonic antigen (CEA), CD19, CD22, and human epidermal growth factor receptor 2 (HER2). Additional targets comprise mesothelin (MSLN), glypican-3 (GPC3), vascular endothelial growth factor receptor 2 (VEGFR2), and epidermal growth factor receptor (EGFR). Other identified antigens include CD47, disialoganglioside GD2, and chondroitin sulfate proteoglycan 4 (CSPG4) [155]. Accumulating evidence indicates that the anti-tumor activity of CAR-M is largely dependent on its phagocytic capacity. Three distinct mechanisms of tumor cell internalization have been described: complete engulfment, trogocytosis, and efferocytosis [155].

TAM-targeting approaches are also being explored in combination with tumor vaccination strategies, although efficacy in solid tumors is frequently constrained by immunosuppressive myeloid cell accumulation within the TME [140]. Tumor vaccination has been shown to promote the expansion of CD11b⁺ immunosuppressive myeloid cells, thereby driving resistance to immunotherapy [141, 142]. Depletion of these cells using anti-CD11b antibodies synergized with vaccination to prolong survival in tumor-bearing mice, despite minimal effects on tumor burden [143]. Similarly, tumor lysate–pulsed dendritic cell vaccines extended survival in murine models, an effect further enhanced by PLX3397, a CSF-1R inhibitor that reprograms macrophages [142].

Oncolytic viruses represent a promising immunotherapeutic modality for multiple cancers [144–147], and TAMs are increasingly recognized as critical regulators of virotherapy efficacy. The impact of TAMs is tumor-model dependent: M1-like macrophages often support tumor regression despite accelerating viral clearance, whereas M2-like macrophages antagonize therapy by promoting tumor growth and suppressing immune responses [148]. Several studies report that oncolytic virus therapy increases the proportion of pro-inflammatory macrophages within the TME, shifting TAMs toward an immunostimulatory phenotype [149–151]. While this pro-inflammatory shift enhances antitumor immunity, it can also accelerate antiviral immune responses and limit viral persistence, highlighting the dual and context-dependent interaction between oncolytic viruses and TAMs [148].

**Fig. 2:**
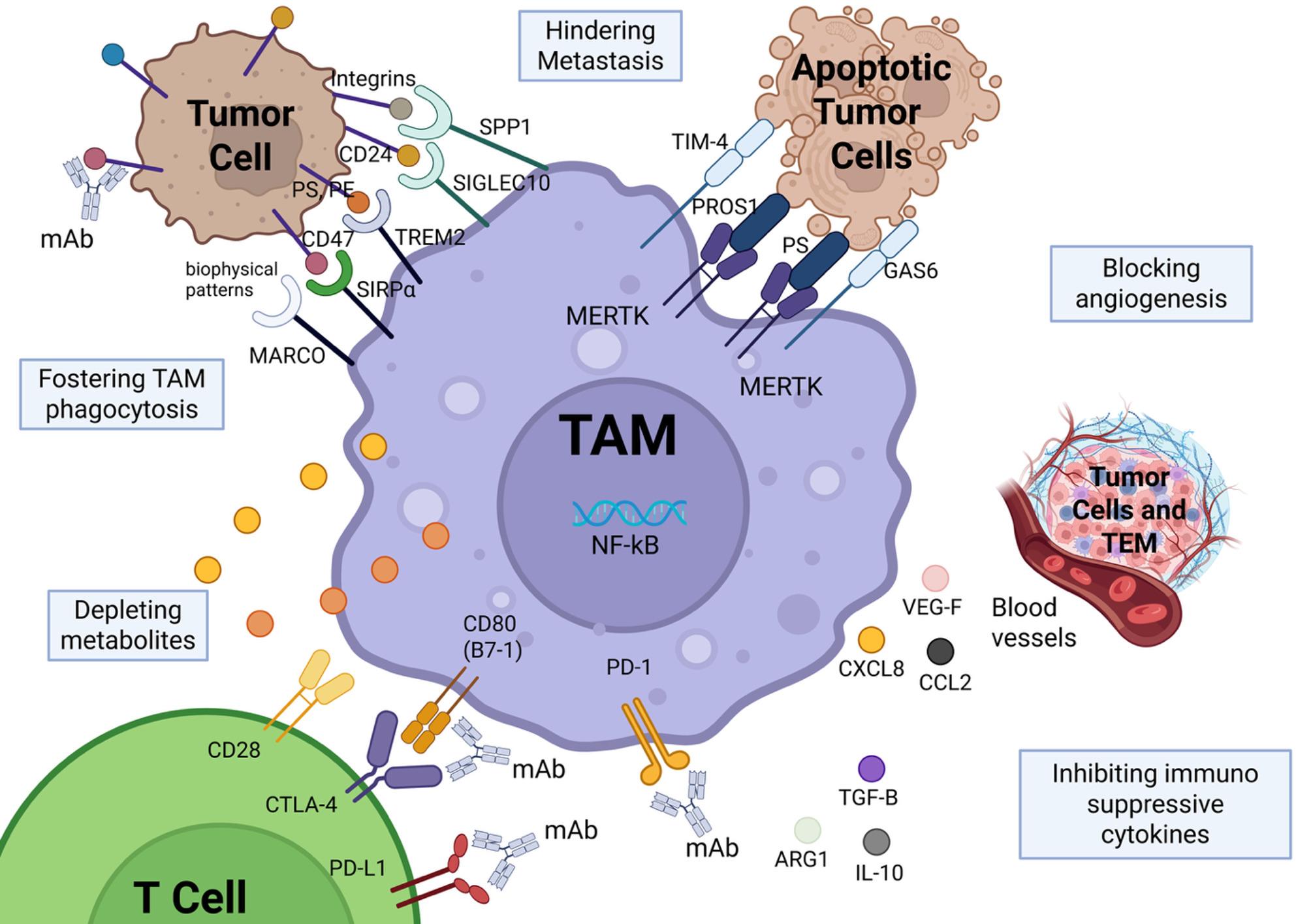
Therapeutic targeting of tumor-associated macrophage (TAM) to induce cancer immune suppression. Diagram of therapeutic strategies directed at reprogramming or inhibiting the immunosuppressive TAM functions in the microenvironment. Targeting pathways associated with TAM can slow tumor progression by reducing immune suppression, therefore hindering metastasis and angiogenesis, thereby enhancing an exquisite antitumor immunity. Strategies include disrupting inhibitory receptor-ligand interactions between TAMs and tumor or immune cells, promoting TAM phagocytosis, hindering immune suppressive cytokines, depleting tumor-supportive metabolites

Collectively, TAMs constitute a dominant and highly heterogeneous immune population within the TME, exerting context-dependent effects on tumor immunity and immunotherapeutic outcomes. Current evidence supports three overarching TAM-targeting strategies—TAM elimination, inhibition of TAM recruitment, and functional reprogramming—which have demonstrated efficacy in preclinical models and, in some cases, progressed to clinical evaluation as adjuvants to immunotherapy [115] (Fig. 2).

## Therapeutic strategies targeting TAMs

**Fig. 3:**
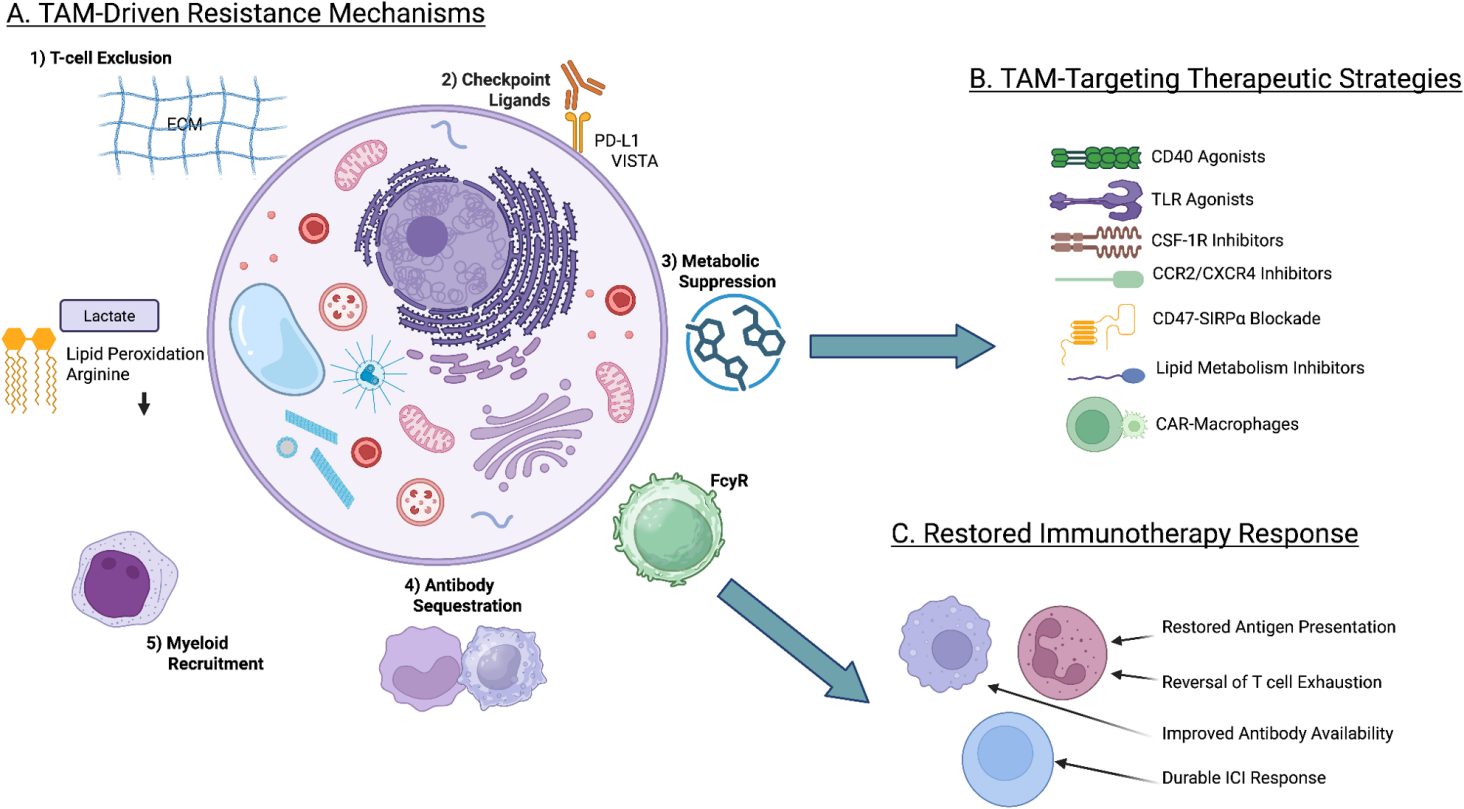
TAM mechanisms of resistance to immunotherapy and strategies to overcome them. **A** TAMs promote resistance to ICI through multiple ways: physical exclusion of T cells, expression of inhibitory checkpoint ligands, metabolic suppression driven by hypoxia, lactate and lipid peroxidation, Fcγ receptor–mediated sequestration of therapeutic antibodies, and compensatory recruitment of immunosuppressive myeloid populations. These mechanisms collectively stabilize T-cell exhaustion and limit reinvigoration under PD-1/PD-L1 blockade. **B** TAM therapeutic strategies: macrophage reprogramming (CD40 and TLR agonists, PI3K-γ inhibition), depletion (CSF-1R blockade), inhibition of monocyte recruitment and retention (CCR2, CXCR2, CXCR4), metabolic reprogramming, macrophage immune checkpoint blockade (CD47–SIRPα), FcγR modulation, and latest cell-based approaches, such as CAR-macrophages. **C** Combinational strategies integrate TAM-targeting agents with immune checkpoint inhibitors to remodel the tumor microenvironment, restore antigen presentation, reverse T-cell exhaustion, and enhance durable antitumor immunity. Abbreviations: TAMs, tumor-associated macrophages; ICI, immune checkpoint inhibitor; ECM, extracellular matrix

The recognition of TAMs as central players in tumor progression and therapy resistance has catalyzed the development of numerous therapeutic strategies. These approaches can be broadly categorized into four key modalities: (1) reprogramming TAMs to an antitumor phenotype, (2) depleting TAMs from the TME, (3) blocking monocyte recruitment, and (4) combination therapies. Current research on these approaches is summarized in Table 2 (Fig. 3).

**Table 2:** Therapeutic strategies targeting Tumor-Associated Macrophages (TAMs) and relevance to immunotherapy resistance

Strategy Class	Representative Targets / Agents	Primary Mechanism of Action	Effect on Immunotherapy Resistance	Preclinical / Clinical Status
TAM reprogramming (activation)	CD40 agonists (selicrelumab, sotigalimab, mitazalimab)	Induce M1-like polarization and enhance antigen presentation	Converts immune-cold tumors to immune-hot; restores T-cell priming and ICI responsiveness	Preclinical; early clinical trials
Pattern recognition receptor activation	TLR3/7/8/9 agonists (poly(I: C), imiquimod, resiquimod, CpG-ODNs)	Activate NF-κB and type I IFN signaling in TAMs	Enhances T-cell infiltration and synergizes with ICIs	Preclinical; early clinical
PI3K-γ inhibition	IPI-549, TG100-115	Blocks immunosuppressive PI3K-γ signaling in myeloid cells	Reverses TAM-mediated T-cell exclusion and exhaustion	Preclinical; clinical
Metabolic reprogramming of TAMs	FAO inhibitors, CPT1A inhibitors, lipid metabolism modulators	Disrupt lipid-driven suppressive TAM phenotypes	Reduces metabolic suppression and stabilizes T-cell reinvigoration under ICIs	Preclinical
TAM depletion	CSF-1R inhibitors (pexidartinib, BLZ945, emactuzumab)	Reduce TAM survival and differentiation	Partial relief of immunosuppression; limited efficacy as monotherapy	Clinical trials (mixed outcomes)
Monocyte recruitment blockade	CCR2 antagonists (PF-04136309), CCL2 antibodies	Prevent monocyte trafficking into tumors	Limits replenishment of suppressive TAMs	Preclinical; early clinical
Myeloid trafficking / retention blockade	CXCR4 inhibitors (plerixafor), CXCR2 antagonists	Disrupt TAM and neutrophil localization within tumors	Improves T-cell infiltration and sensitizes tumors to ICIs	Preclinical; clinical
Macrophage immune checkpoint blockade	CD47–SIRPα inhibitors (magrolimab, Hu5F9-G4)	Restores macrophage-mediated phagocytosis	Enhances antigen release and T-cell priming; synergizes with ICIs	Clinical trials
FcγR modulation	Fc-engineered antibodies, FcγR blockade strategies	Prevents TAM-mediated antibody sequestration	Improves ICI bioavailability and efficacy	Preclinical
Nanoparticle-based TAM targeting	TLR-agonist or drug-loaded nanoparticles	Selective TAM reprogramming with reduced systemic toxicity	Overcomes macrophage-driven resistance niches	Preclinical
Cell-based therapies	CAR-macrophages (CT-0508)	Direct tumor phagocytosis and TME remodeling	Bypasses T-cell exclusion and myeloid suppression	Phase I clinical trials
Combination strategies	TAM-targeting agents + ICIs / chemotherapy / radiotherapy	Multi-axis immune reactivation	Prevents compensatory resistance mechanisms	Preclinical; clinical

In addition, the preclinical and clinical research on strategies targeting TAMs is listed in Table 3. Although the preclinical rational is strong, limited is the clinical efficacy observed by TAM-targeting agents— such as CSF-1/CSF-1R inhibitors monotherapies in clinical trials. This could be easily attributed to several mechanisms for TAMs. First, CSF-1R blockade frequently induces compensatory recruitment of alternative immunosuppressive myeloid populations, including CCR2⁺ monocytes, CXCR4⁺ macrophages, and neutrophils, which rapidly restore immune suppression [167]. Second, there is an incomplete depletion of TAMs or based only on certain subsets. Macrophages and TAM subsets develop resistance to CSF-1R inhibitors [168]. Third, because of systemic toxicities associated with CSF-1R inhibition, including hepatotoxicity and edema, there are limits to dosing intensity and treatment duration, constraining therapeutic efficacy overall [169, 170]. Finally, because of redundant suppressive pathways and defective T-cell trafficking TAM depletion alone is insufficient to reinvigorate antitumor immunity [171]. Collectively, these limitations explain why TAM-targeting agents have shown modest activity as monotherapies, providing a strong rationale for combination strategies integrating TAM modulation with immune checkpoint blockade or innate immune activation or other targeted therapies.

**Table 3:** Preclinical and clinical research on strategies targeting Tumor-Associated Macrophages (TAMs)

Therapeutic Strategy	Mechanism of Action	Preclinical/Clinical Status	Reference
CSF-1/CSF-1R blockade	Improves response to immune checkpoint inhibitors and adoptive cell therapy	Preclinical	[156, 157]
CD40 agonist + anti–CSF-1R antibodies	Reprograms TAMs toward M1 before depletion; induces T cell–mediated antitumor immunity	Preclinical	[158, 159]
PI3K-γ inhibition	Blocks immunosuppressive signaling and activates proinflammatory programs in TAMs	Preclinical	[138]
Chimeric antigen receptor macrophages (CT-0508)	Chimeric antigen receptor macrophages directly kill tumor cells and remodel the tumor microenvironment	Phase I trial (NCT04660929)	[160]
Oncolytic parvovirus (H-1PV) + IFN-γ	Enhances macrophage antitumor activity and immune infiltration	Preclinical	[161]
Oncolytic adenovirus (OAd–TNF-α–IL-2) + chimeric antigen receptor T cells	Induces M1 polarization and dendritic cell maturation; enhances T-cell and chimeric antigen receptor T-cell infiltration	Preclinical	[162]
Oncolytic adenovirus (TMZ-CD40L)	Expresses trimerized CD40L; drives TAM M1 polarization; increases tumor-infiltrating lymphocytes and promotes antitumor immunity	Preclinical	[163]
Nanoparticle–TLR agonist delivery (e.g., R848)	Targets TAMs to reeducate them and present antigens; converts resistant tumors to be responsive to adaptive immune-cell transfer	Preclinical	[46, 138, 164]
Oncolytic HSV + immune checkpoint inhibitors (anti–CTLA-4, anti–PD-1) + IL-2	Promotes macrophage influx and M1 reprogramming	Preclinical	[161]
Oncolytic adenovirus (DNX-2401)	Replicates in Rb-defective glioma; activates M1 macrophages; induces cytokine production and M1 markers in patients	Phase I/II (NCT01582516)	[150]
Integrin β1 inhibition	Blocks TAM migration and enhances oncolytic virus replication and efficacy	Preclinical	[165]
IL-13–expressing oncolytic adenovirus	Reduces M2 TAMs; boosts antiviral oncolysis	Preclinical	[166]

### TAM reprogramming

Rather than depleting macrophages, a more nuanced strategy is to “re-educate” or reprogram the immunosuppressive, pro-tumoral TAMs into immunostimulatory, antitumoral effector cells. This approach aims to shift the balance within the TME from pro-tumor to antitumor without compromising the potential beneficial functions of macrophages.

#### CD40 agonists

CD40 is a member of the TNF receptor superfamily expressed on antigen-presenting cells, including macrophages, dendritic cells, B cells, and NK cells [172]. Its natural ligand (CD40L) is typically expressed on activated CD4 + T cells, providing a critical signal for T-cell priming and antigen-presenting cell activation. Agonistic anti-CD40 antibodies mimic this signal. Macrophages polarized to the anti-tumor phenotype facilitate Th1 immune reactions. These responses are characterized by the identification and eradication of malignant cells, coupled with the presentation of tumor-associated antigens to T lymphocytes. This immunostimulatory function is mediated through the secretion of pro-inflammatory cytokines, IL-1β, IL-6, and TNF-α [173]. anti-tumor macrophages directly induce programmed cell death pathways in cancer cells, including apoptosis and ferroptosis, primarily via the generation of reactive oxygen species and nitric oxide. Additionally, anti-tumor macrophages trigger pyroptosis in tumor cells [49]. Conversely, the pro-tumorigenic macrophage phenotype exerts immunosuppressive effects that attenuate antitumor activity and promote Th2 responses. This is achieved through the release of cytokines such as IL-10, IL-13, and TGF-β. Within the TME, TAMs are predominantly skewed toward an M2-polarized state. This M2-like polarization is a critical facilitator of oncogenesis and contributes to all stages of malignant progression [173].

Engagement of CD40 with its natural ligand (CD40L or CD154) or with agonistic antibodies triggers a cascade of intracellular signaling events that profoundly reprogram macrophage phenotype and function. The TRAF adaptor proteins serve as key mediators of CD40 signaling by triggering multiple downstream cascades, including the canonical and non-canonical NF-κB pathways, mitogen-activated protein kinases (MAPKs), PI3K, and phospholipase Cγ (PLCγ) signaling. Following interaction with CD40L, the cytoplasmic tail of CD40 facilitates the direct or indirect recruitment of adaptor proteins, including TRAF1, TRAF2, TRAF3, TRAF5, and TRAF6 [174, 175]. Through CD40 signaling, both the canonical and non-canonical NF-κB pathways can be activated [176, 177]. NF-κB is a central transcription factor that drives anti-tumor polarization and the expression of inflammatory mediators [178]. TRAF2, TRAF3, and TRAF5 bind to CD40 and play a key role in triggering the non-canonical NF-κB pathway. Engagement of CD40 with either TRAF2 or TRAF3 alone can also support canonical NF-κB activation, whereas activation of the non-canonical pathway requires the simultaneous involvement of both TRAF2 and TRAF3. Additionally, recruitment of TRAF6 to CD40 specifically drives the canonical NF-κB pathway [177].

CD40 agonist therapy can induce tumor regression through immune mechanisms that are either dependent on or independent of T lymphocytes. The T-cell–independent pathway involves modulating the local TME, primarily through the activation of macrophages, which leads to the destruction of the tumor’s stromal architecture. In contrast, the T-cell–dependent pathway functions by enhancing antigen presentation capabilities and directly promoting the activation of cytotoxic T cells [173].

Several agonists of CD40 have been developed and investigated for their potential to reprogram TAMs and enhance antitumor immunity. Among them, selicrelumab (CP-870,893), a fully human agonist antibody, has been tested in clinical trials for pancreatic cancer and melanoma, where it activated TAMs, induced inflammatory cytokine production, and promoted T-cell–mediated responses [179, 180]. CP-870,893 induces apoptosis in CD40-expressing tumor cells. In pancreatic cancer, in combination with gemcitabine and cobimetinib, it shows successful efficacy in overcoming resistance to conventional therapies [49]. Mitazalimab (ADC-1013; JNJ-64457107), a human anti-CD40 agonist IgG1 antibody, demonstrated preclinical and early clinical efficacy by repolarizing TAMs toward an M1-like phenotype and enhancing antigen presentation and expansion-specific T-cell response [181, 182]. Sotigalimab (APX005M) is another humanized IgG1 monoclonal antibody targeting CD40. Sotigalimab is anticipated to potentiate innate and adaptive immune responses, facilitate activation of antigen-presenting cells for optimal antigen processing and T-cell priming, enhance antitumor T-cell activity, modulate the TME via reprogramming of TAMs, and induce the transition of immunologically “cold” tumors into “hot” tumors. A previous study showed that the combination of sotigalimab with nivolumab produced long-lasting responses in some patients with melanoma resistant to anti–PD-1 therapy [183]. Furthermore, cifurtilimab (SEA-CD40), a non-fucosylated, humanized monoclonal IgG1 antibody that stimulates CD40, is currently in phase I trials and has been reported to promote antitumor immune activation [184].

#### TLR agonists

Toll-like receptors (TLRs) are found on innate immune cells, including dendritic cells and macrophages, as well as on non-immune cells like fibroblasts and epithelial cells. Activation of TLR signaling pathways promotes MyD88, TRIF, and NF-κB, which enhance pro-inflammatory cytokine production and antigen presentation [185], thereby shifting TAMs toward an M1-like phenotype. Recent studies have shown that TLR agonists, such as poly(I: C) (TLR3), monophosphoryl lipid A and BCG (TLR2, TLR4), imiquimod and resiquimod (TLR7/8), and CpG oligodeoxynucleotides (TLR9), can effectively reprogram TAMs, restore antitumor immunity, and enhance the efficacy of immunotherapies [164, 186, 187].

Polyinosinic-polycytidylic acid [poly(I: C)], a synthetic double-stranded RNA, is a well-characterized TLR3 agonist that stimulates TRIF-dependent signaling, leading to activation of IRF3 and NF-κB pathways. This results in the production of type I IFN (IFN-α/β) and pro-inflammatory cytokines, which drive TAM polarization toward an anti-tumor phenotype, decrease the frequency of MDSCs, and enhance cytotoxic T-cell responses [188–190].

The TLR4 agonist monophosphoryl lipid A, a detoxified derivative of lipopolysaccharide, has been widely used as a vaccine adjuvant. In cancer models, monophosphoryl lipid A and IFN-γ stimulate macrophages to upregulate costimulatory molecules (CD80, CD86) and secrete inflammatory cytokines, thereby facilitating antitumor T-cell activation. TLR4 activation also promotes the release of reactive oxygen and nitrogen species, enhancing the direct tumoricidal activity of macrophages [191, 192].

Imiquimod and resiquimod (R848) are small-molecule agonists that activate TLR7 and TLR8, leading to MyD88-dependent activation of NF-κB and IRF3/7. These agonists stimulate TAMs to produce IL-12, TNF-α, and type I interferons, promoting M1-like polarization [164, 186, 193, 194]. Topical imiquimod is already FDA-approved for superficial basal cell carcinoma, demonstrating the translational potential of TLR7 agonists in oncology [156].

Synthetic CpG oligodeoxynucleotides (CpG-ODNs) mimic bacterial DNA and activate TLR9 signaling [195]. Engagement of TLR9 reprograms TAMs to secrete pro-inflammatory cytokines and enhance antigen presentation [196, 197]. Previous studies have shown that CpG-ODNs can synergize with ICIs (anti–PD-1 antibody), thereby overcoming resistance to PD-1/PD-L1 blockade and restoring effective T-cell responses [197]. Several TLR9 agonists are currently under clinical investigation for various malignancies. Notable examples include IMO-2055 and CpG-7909 for advanced non–small cell lung cancer; dSLIM for advanced colorectal cancer; MGN1703 for small cell lung cancer and solid tumors; and SD-101 for follicular lymphoma. Investigations of other agents, such as KSK-CpG for melanoma, ODN M362 for hepatocellular carcinoma, and CpG-1826 (which has shown efficacy in glioma xenografts), further demonstrate the broad therapeutic potential of this class of compounds [198].

#### PI3K-γ inhibitors

PI3Ks, which are part of the lipid kinase family, are essential in regulating various immune cell functions like activation, proliferation, and differentiation. Emerging evidence underscores the PI3K signaling pathway as a key regulator of both tumor growth and the immune microenvironment. By enhancing the activity of Tregs and MDSCs, this pathway creates an immunosuppressive environment within tumors, which reduces the effectiveness of cancer vaccines and immunotherapies [199]. Class I PI3Ks are divided into class IA (including PI3K-α, PI3K-β, and PI3K-δ) and class IB (represented by PI3K-γ). PI3K-α and PI3K-β are widely found in many cell types, such as epithelial cells, whereas PI3K-δ is mainly limited to T lymphocytes, and PI3K-γ is found only in myeloid cells. The activation of PI3K-γ enhances signaling networks linked to immune suppression [200]. Targeting PI3K-γ has become a promising approach to reprogram the immunosuppressive TME. Selective PI3K-γ inhibitors can reverse the M2-like polarization of TAMs, shifting them to an immunostimulatory M1-like state. This change in the myeloid cells helps overcome a major resistance mechanism to ICIs, resulting in stronger antitumor T-cell responses and better synergy with anti–PD-1/PD-L1 therapies. IPI-549 and TG100-115 are the main PI3K-γ inhibitors, both exhibiting high selectivity toward the gamma isoform [200].

#### Other TAM reprogramming agents

Compounds such as BLZ945, PLX3397, and pexidartinib that block the CSF-1R receptor do not deplete TAMs but instead shift their profile from pro-tumorigenic to anti-tumor, leading to downregulation of pro-tumor genes and improved survival in animal models of glioblastoma and breast tumors. When combined with ICIs, CSF-1R antagonists boost tumor infiltration and activity by killing T cells within tumors, thus surmounting immunosuppressive obstacles within tumors [201–203].

Discovered just a few years ago, a new target for macrophage reprogramming is the scavenger receptor MARCO (macrophage receptor with collagenous structure), which is abundantly expressed on TAMs across multiple solid tumor types. Inhibition of MARCO using monoclonal antibodies such as ED31 has been shown to shift TAMs toward a pro-inflammatory M1-like phenotype without eliminating them. This phenotypic switch increases the production of M1-associated chemokines (including CCL2, CCL3, CCL4, CCL5, CXCL1, CXCL10, and CCL22), thereby enhancing the recruitment of immune effector cells such as CD8 + T cells, NK cells, and conventional dendritic cells into the TME. Interestingly, MARCO inhibition exhibits a synergistic effect with anti–CTLA-4 therapy, but not with anti–PD-1 therapy, resulting in enhanced tumor suppression and prolonged survival in mouse tumor models [204].

Additionally, bispecific antibodies targeting CD47–SIRPα enhance phagocytosis and suppress the immunosuppressive activity of TAMs. The interaction between SIRPα and CD47 represents a promising therapeutic target. SIRPα, which is highly expressed on macrophages, binds to CD47, a molecule overexpressed on cancer cells. This interaction transmits a “don’t eat me” signal that suppresses macrophage-mediated phagocytosis, thereby allowing tumor cells to evade immune recognition and destruction. Consequently, disruption of the CD47–SIRPα signaling axis has shown potent antitumor effects by enhancing macrophage phagocytosis, improving antigen presentation, and inhibiting tumor progression [205]. In this regard, Hu5F9-G4 and magrolimab have shown promise in preclinical cancer research, and their combination with radiotherapy or chemotherapy agents like temozolomide leads to highly effective anticancer responses without detriment to healthy neural tissue [83, 161, 206, 207].

Although tumor-promoting pro-tumorigenic macrophages typically show high oxidative phosphorylation (OXPHOS) levels, a recent study found that CD40 signaling can repurpose fatty acid oxidation. Instead of supporting the pro-tumorigenic state, the acetyl-CoA produced by fatty acid oxidation helps activate pro-inflammatory and antitumor genes. Inhibitors such as carnitine palmitoyltransferase 1 A (CPT1A) blockers and IACS-010759 preferentially eliminate pro-tumorigenic cells [208].

### TAM depletion

An alternative or complementary strategy to macrophage reprogramming is the direct depletion of TAMs, or ablation [209]. Several methods can be used in this approach.

Antibodies (e.g., lacnotuzumab) target the CSF-1/CSF-1R axis, a critical pathway for the survival, proliferation, and differentiation of most tissue-resident macrophages and TAMs. By inhibiting CSF-1R or blocking its ligand, these therapies can effectively reduce TAM density in the TME. Preclinical studies have shown that depleting immunosuppressive TAMs can enhance T-cell infiltration and synergize with ICIs [209]. However, clinical outcomes have been mixed, highlighting the complexity of myeloid targeting and the potential need for patient stratification based on TAM density.

Engineered CAR T cells (F4.CAR-T) are an emerging innovation that has been reprogrammed to recognize macrophage-specific antigens (F4/80) within the TME. In vitro studies in ovarian, pancreatic, and lung cancer have shown that macrophage-targeting CAR T cells efficiently deplete TAMs and induce robust antitumor immunity [210].

RNA-based strategies, leveraging small activating RNAs and circular RNAs, introduce new possibilities for modulating macrophage regulatory circuits and boosting their anticancer effectiveness. Drug conjugates tailored for macrophage delivery and nanoparticle systems provide increased precision and lower toxicity by selectively targeting TAMs [194, 211].

### Blocking monocyte recruitment

The CCR2/CCL2 signaling axis is a major pathway that regulates the recruitment of monocytes into the TME, where they differentiate into TAMs that support tumor growth and immune evasion. CCL2 (C–C motif chemokine ligand 2), secreted by tumor and stromal cells, binds to its receptor CCR2 (C–C motif chemokine receptor 2) on circulating monocytes, driving their infiltration into tumor tissues and subsequent polarization toward an M2-like, immunosuppressive phenotype. Inhibition of this axis has therefore emerged as a promising therapeutic strategy to prevent monocyte recruitment and limit TAM accumulation [212]. CCR2/CCL2 inhibitors include neutralizing antibodies against CCL2, such as carlumab (CNTO 888), and small-molecule CCR2 antagonists, including PF-04136309 and CCX872. Preclinical and early clinical studies have shown that blocking the CCR2/CCL2 pathway decreases TAM infiltration [212].

The CXCR2 signaling pathway plays a crucial role in recruiting neutrophils and MDSCs to the TME, where they contribute to immunosuppression and promote tumor progression [213]. CXCR2 antagonists (e.g., SB225002, SCH-527123, AZD5069, SCH-479833, ladarixin [DF2156A], and reparixin) inhibit the CXCL1-CXCR2 axis, thereby reducing the infiltration of neutrophil N2, which cooperates with TAM and leads to T-cell–mediated antitumor immunity [214].

### Combination therapies

Combining TAM-targeted therapy with radiotherapy or chemotherapy has emerged as an effective strategy to enhance antitumor immunity. Radiotherapy and certain chemotherapeutic agents can induce immunogenic cell death, leading to the release of tumor-associated antigens and danger signals that stimulate dendritic cells and T-cell activation. However, these treatments also promote the recruitment and polarization of TAMs toward an M2-like, immunosuppressive phenotype, which can limit therapeutic efficacy. Chemotherapeutic agents, immunotherapies, and radiotherapy at moderate doses have shown the capacity to alter the TME by reprogramming TAMs. This process shifts their functional state from a pro-tumorigenic phenotype to an anti-tumor phenotype, which can enhance immune-mediated tumor cell killing. Conversely, resistance to chemotherapy (cisplatin and carboplatin) in ovarian and cervical cancer cell lines has been observed with increased production of PGE2 and IL-6 by tumor cells and a shift of monocyte polarization toward the pro-tumorigenic phenotype [215]. TAM-targeted agents can counteract this effect by depleting or reprogramming suppressive macrophages, thereby amplifying the immunostimulatory impact of radiotherapy or chemotherapy and improving tumor control [216]. In fact, combinatorial strategies integrating chemotherapy with immunotherapeutic agents have resulted in augmented representation of M1-polarized TAMs. This shift is characterized by elevated expression of M1-associated markers and concomitant suppression of M2-related factors.

## Discussion and future perspectives

Over the past decade, TAMs have emerged as a central player linking inflammation, immune suppression, and therapy resistance. Yet their inherent plasticity and adaptability make them both a challenge and an opportunity for next-generation cancer therapy.

Mapping TREM2, SPP1, CD163, and IFN-γ populations across tumor types and treatment contexts will allow more precise, patient-tailored strategies. Therapeutically, targeting TAMs represents a promising way to overcome resistance and enhance immunotherapy efficacy. Strategies are evolving beyond simple macrophage depletion toward more sophisticated reprogramming approaches that shift TAMs from a pro-tumoral to an antitumoral phenotype. Inhibitors of CSF-1R, CCR2, or CXCR4 can reduce immunosuppressive macrophage accumulation, whereas agents activating CD40, TLRs, or STING pathways can re-educate the immune system to promote an inflammatory state. Recently, the combination of macrophage modulators with PD-1/PD-L1 blockade led to synergistic effects, increasing T-cell infiltration and improving tumor control in preclinical models [217, 218].

Additionally, Early-phase trials of HER2-targeted CAR-M in solid tumors have shown encouraging safety and early signs of immune activation [219, 220]. The combination of CAR-M therapy with ICIs or oncolytic viruses could represent a new frontier in immunotherapy for refractory solid tumors. CAR-M technologies have rapidly progressed from preclinical proof-of-concept to early clinical evaluation, highlighting the translational potential of macrophage-based cell therapies in solid tumors. CAR-M platforms exploit the innate tumor-infiltrating and phagocytic capabilities of macrophages by equipping them with antigen-specific receptors, thereby enhancing direct tumor cell engulfment, pro-inflammatory cytokine production, and remodeling of the immunosuppressive tumor microenvironment [221]. In preclinical models, CAR-M exhibited antigen-specific phagocytosis, induction of pro-inflammatory programs, conversion of bystander M2 macrophages toward M1 phenotypes, upregulation of antigen presentation machinery, and recruitment of adaptive immune cells, leading to reduced tumor burden and prolonged survival [157]. Moreover, CAR-M has demonstrated the capacity to synergize with chemotherapy and checkpoint blockade, further amplifying antitumor responses and overcoming resistance mechanisms in solid tumor models [158].

Clinically, multiple CAR-M constructs, including anti-HER2 CAR-M, have entered phase I trials, showing feasibility, manageable safety profiles, and evidence of tumor trafficking and microenvironment modulation, such as increased T cell infiltration and antigen spreading in treated patients [219]. Additionally, emerging CAR-M targeting c-MET in pancreatic cancer models revealed enhanced tumor infiltration, potent phagocytic activity, and synergistic effects with cytotoxic chemotherapy without severe toxicities, underscoring their relevance in traditionally refractory malignancies [159].

Despite these advances, significant challenges remain, including improving in vivo persistence of CAR-M cells, optimizing gene transduction methodologies, and developing scalable manufacturing platforms that maintain pro-inflammatory phenotypes without inducing off-target effects or macrophage exhaustion [157]. Addressing these hurdles, alongside strategic combinations with immune-checkpoint inhibitors and metabolic modulators, will be crucial for translating CAR-Mtherapies into durable and broadly effective treatments for solid tumors.

Furthermore, metabolic and epigenetic reprogramming of TAMs to remove macrophage-mediated immunosuppression has gained attention. Modulating these pathways, for instance, through inhibition of arginase-1, IDO, or lactate dehydrogenase, may restore macrophage function and reinvigorate antitumor immunity [222]. Likewise, targeting epigenetic regulators such as histone deacetylases, BET proteins, or DNA methyltransferases could reverse stable suppressive states, making TAMs more amenable to immune activation [223].

Noninvasive imaging of TAMs is another emerging area with transformative potential. Novel radiotracers for PET imaging, such as those targeting TSPO or CD206, can help visualize macrophage distribution and monitor treatment response in real time [160]. These imaging biomarkers may enable early identification of patients likely to benefit from TAM-targeted therapies and facilitate adaptive treatment strategies.

Moreover, there is growing recognition that macrophage–T-cell crosstalk is central to both resistance and response. TAMs can directly inhibit cytotoxic T lymphocytes via checkpoint ligands, cytokines, and metabolic competition. Conversely, IFN-γ produced by activated T cells can remodel macrophage polarization [162]. Understanding these bidirectional interactions enhances the rationale for combination therapies, where modulating macrophage phenotypes restores T-cell function and amplifies the impact of checkpoint inhibition.

Several challenges remain. TAM-targeting therapies must achieve a delicate balance between reducing immunosuppressive functions and preserving tissue homeostasis [171]. Besides, the context-dependent nature of macrophage signaling suggests that timing, dosage, and tumor subtype will critically determine therapeutic success. Integrating computational modeling, systems immunology, and longitudinal sampling will be key to unraveling these complexities. In the coming years, the convergence of multi-omics profiling, advanced imaging, and precision immunotherapy will likely redefine how TAMs are viewed and targeted. Instead of being treated as a uniform suppressive population, macrophages will be understood as dynamic and context-specific immune regulators. Combining macrophage-modulating agents with ICIs, adoptive T-cell therapy, or tumor vaccines could convert resistant “cold” tumors into inflamed “hot” ones capable of sustaining durable immune control [168].

Ultimately, the future of cancer immunotherapy will depend not only on empowering T cells but also on re-educating the myeloid compartment to support effective and lasting antitumor immunity. By bridging insights from basic macrophage biology with clinical innovation, it may be possible to overcome current resistance mechanisms and extend the transformative benefits of immunotherapy to a broader patient population.

## Conclusion

TAMs are pivotal regulators of immunotherapy resistance. Their heterogeneity, plasticity, and strategic localization within the TME make them a formidable barrier to effective treatment. Therapeutic strategies that deplete, reprogram, or block recruitment of TAMs—particularly in combination with ICIs or cellular therapies—hold promise for overcoming resistance. Future research integrating mechanistic studies, biomarker development, and rational combination strategies is critical to fully leverage the potential of immunotherapy in solid tumors.

## Data Availability

All data and materials are included in the references.

## Abbreviations

ARG1: Arginase-1
CAR-M: Chimeric antigen receptor macrophages
CAR-T: Chimeric Antigen Receptor–T-Cell
CpG-ODNs: CpG Oligodeoxynucleotides
CSF: Colony-Stimulating Factor
CTLA-4: Cytotoxic T-Lymphocyte–Associated Protein-4
ECM: Extracellular Matrix
HIF: Hypoxia-Inducible Factors
ICIs: Immune Checkpoint Inhibitors
IDO: Indoleamine 2,3-Dioxygenase
MARCO: Macrophage Receptor with Collagenous Structure
MDSCs: Myeloid-Derived Suppressor Cells
MMP: Matrix Metalloproteinases
PD-1: Programmed Cell Death Protein-1
PDGFβ: Platelet-Derived Growth Factor β
RGMb: Repulsive Guidance Molecule b
SIRPα: Signal Regulatory Protein ɑ
TAMs: Tumor-Associated Macrophages
TGF: Transforming Growth Factor
TME: Tumor Microenvironment
